# Immune response after intermittent minimally invasive intraocular pressure elevations in an experimental animal model of glaucoma

**DOI:** 10.1186/s12974-016-0542-6

**Published:** 2016-04-18

**Authors:** Oliver W. Gramlich, Julia Teister, Mareike Neumann, Xue Tao, Sabine Beck, Harald D. von Pein, Norbert Pfeiffer, Franz H. Grus

**Affiliations:** Experimental Ophthalmology, Department of Ophthalmology, University Medical Center of the Johannes Gutenberg University Mainz, Langenbeckstr. 1, 55131 Mainz, Germany; Glaucoma Cell Biology Laboratory, Department of Ophthalmology and Visual Sciences, The University of Iowa, Iowa City, IA 62242 USA; Department of Neuropathology, University Medical Center of the Johannes Gutenberg University Mainz, Langenbeckstr. 1, Mainz, 55131 Germany

**Keywords:** Glaucoma, Ocular hypertension, Pressure fluctuations, Neuronal degeneration, Retinal ganglion cells, Humoral immune system, Autoantibodies, B lymphocyte inhibitor, Belimumab

## Abstract

**Background:**

Elevated intraocular pressure (IOP), as well as fluctuations in IOP, is a main risk factor for glaucoma, but its pathogenic effect has not yet been clarified. Beyond the multifactorial pathology of the disease, autoimmune mechanisms seem to be linked to retinal ganglion cell (RGC) death. This study aimed to identify if intermittent IOP elevations in vivo (i) elicit neurodegeneration, (ii) provokes an immune response and (iii) whether progression of RGC loss can be attenuated by the B lymphocyte inhibitor Belimumab.

**Methods:**

Using an intermittent ocular hypertension model (iOHT), Long Evans rats (*n* = 21) underwent 27 unilateral simulations of a fluctuating pressure profile. Nine of these animals received Belimumab, and additional seven rats served as normotensive controls. Axonal density was analyzed in PPD-stained optic nerve cross-sections. Retinal cross-sections were immunostained against Brn3a, Iba1, and IgG autoantibody depositions. Serum IgG concentration and IgG reactivities were determined using ELISA and protein microarrays. Data was analyzed using ANOVA and Tukey HSD test (unequal N) or student’s independent *t* test by groups.

**Results:**

A wavelike IOP profile led to a significant neurodegeneration of optic nerve axons (−10.6 %, *p* < 0.001) and RGC (−19.5 %, *p* = 0.02) in iOHT eyes compared with fellow eyes. Belimumab-treated animals only showed slightly higher axonal survival and reduced serum IgG concentration (−29 %) after iOHT. Neuroinflammatory events, indicated by significantly upregulated microglia activation and IgG autoantibody depositions, were shown in all injured retinas. Significantly elevated serum autoantibody immunoreactivities against glutathione-S-transferase, spectrin, and transferrin were observed after iOHT and were negatively correlated to the axon density.

**Conclusions:**

Intermittent IOP elevations are sufficient to provoke neurodegeneration in the optic nerve and the retina and elicit changes of IgG autoantibody reactivities. Although the inhibition of B lymphocyte activation failed to ameliorate axonal survival, the correlation between damage and changes in the autoantibody reactivity suggests that autoantibody profiling could be useful as a biomarker for glaucoma.

**Electronic supplementary material:**

The online version of this article (doi:10.1186/s12974-016-0542-6) contains supplementary material, which is available to authorized users.

## Background

Glaucoma is one of the leading causes of blindness worldwide, and the elevation of the intraocular pressure (IOP) is considered a major risk factor [1–3]. However, the pathology of glaucoma is multifactorial and defined as a heterogenic optic neuropathy based on a slow progressive loss of retinal ganglion cells (RGC) [4]. Beside other factors such as vascular dysfunction [5, 6], oxidative stress [7], or retinal glutamate and nitric oxide toxicity [8, 9], inflammatory and autoimmune mechanisms have been demonstrated to play an important role in the pathobiology of RGC loss. These autoimmune mechanisms are characterized by changes of autoantibody patterns in the serum and aqueous humor of glaucoma patients [10–12]. The observation of altered autoantibody profiles in glaucoma is not a unique phenomenon among neurodegenerative diseases, as the humoral immune system has also been implicated in the pathology of Alzheimer’s dementia (AD) and Parkinson’s disease (PD) [13]. Interestingly, AD and PD patients demonstrate pathological changes in the retina and the optic nerve, as well as in other areas of the visual system, leading to impairment of perception, color vision, or contrast sensitivity [14, 15]. Some of the underlying processes of neuronal degeneration, specifically RGC loss in AD as well as PD, share similarities with glaucoma [16, 17]. There are striking parallels between the autoantibody reactions observed in AD, PD, and glaucoma, such as those against glial fibrillary acidic protein (GFAP), S100, and aldolase [11, 18]. However, there is poor understanding of the origin or the primary event that elicits the observed immune response in these neurodegenerative disorders. The overall aim of this study is to investigate whether an immune response, particularly an autoantibody response, is initiated by short-term elevations of the IOP in vivo.

A minimally invasive animal model was recently established to induce intermittent ocular hypertension (iOHT) [19, 20]. This new model was intended to be the least invasive model to increase IOP experimentally in rats and to avoid a strong artificial immune response due to inflammation likely occurring in more invasive approaches, such as the bead occlusion or laser photocoagulation. Using this experimental glaucoma animal model, it is assumed that the predominant changes of the humoral immune system occur in response to the applied pressure and/or to the subsequent loss of RGCs. An increasing number of studies indicate a link between the fluctuation of the IOP in glaucoma patients and disease progression [21, 22]; the advantage of this new model is to mimic these dynamic IOP variations.

If it emerges that changes of the humoral immune response and a loss of RGC can be initiated by a wavelike profile of intermittent ocular hypertensions in vivo, the question of whether or not the subsequent humoral immune response affects the progression of RGC loss can be addressed. Moreover, a pharmaceutical modulation of the B lymphocyte population could have an influence on RGC survival. To test this, Belimumab, an inhibitor of the B lymphocytes activating component of the tumor necrosis factor family (BAFF), was applied to one experimental group before and during IOP elevations to determine the role of the humoral immune system in glaucomatous neurodegeneration.

## Methods

### Animals

Animals were treated according to the ARVO Statement for the Use of Animals in Ophthalmic and Vision Research, and all experiments have been approved by the national investigation office in Koblenz, Germany (23 177-07/G 11-1-029). Eight-week-old male Long Evans rats were obtained from Charles River (Sulzfeld, Germany) and housed in climate-controlled rooms under 12-h light-dark cycles and received food and water ad libitum. Twenty-eight animals were assigned to three groups: CTRL (*n* = 7), iOHT (*n* = 12), and iOHT + Belimumab (*n* = 9).

### Intraocular pressure measurement

In order to detect any additional changes to IOP that may have occurred in response to the manipulations, IOP was monitored routinely in awake animals between 9 am and 12 pm using a TonoLab tonometer (icare, Espoo, Finland). Rats were kept in a horizontal orientation with the head in a relaxed position. No pressure was applied to the head during measurement. The animals became habituated to the IOP measurements quickly. All measurements were performed by the same examiner, and a mean value was calculated from ten consecutive measurements. IOP values that were identified as inaccurate by the TonoLab device were not included in the analysis. The IOP measurements in anesthetized rats were performed immediately before and after onset or adjustment of the loop as well as every five minutes throughout the manipulation. The ΔIOP was defined as the integrated IOP to which the eyes were exposed during the intermittent periods of iOHT. It was calculated from the ∑ of areas under the curve after subtraction of IOP values of the fellow eye.

### Experimental setup

**Fig. 1:**
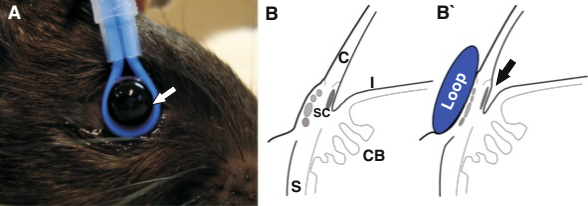
Intermittent ocular hypertension: Induction of short-term elevated intraocular pressure. **a** Eyes of anesthetized Long Evans rats were injured by inducing elevated intraocular pressure levels using a silicone loop around the bulb for 1 h per day for 27 treatments. The loop is attached very gently around the eye without modifying the shape of the eye (*white arrow*) and held in place using a stand. Eyes were kept moist during the time of the experiment. Left eyes served as fellow control eyes. **b** A schematic of the anatomic details of a rodent eye without (**b**) and with the loop attached (**b’**). The loop slightly compresses Schlemm’s canal (SC) as well as the episcleral veins (*in lighter gray*) and provokes a marginal relocation of the iridocorneal angle (indicated by the *arrow*), resulting in an increase of aqueous humor outflow resistance. *Abbreviations*: *C* cornea, *I* iris, *CB* ciliary body, *S* sclera

**Fig. 2:**
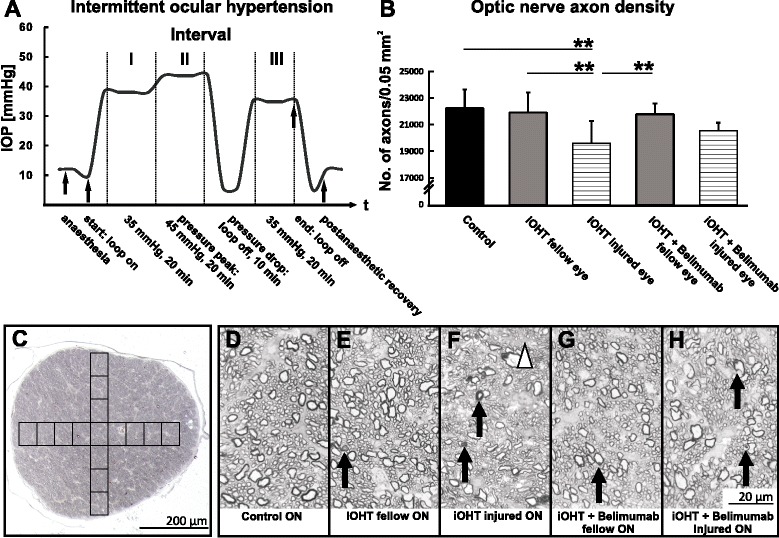
Intermittent intraocular hypertension and analysis of the optic nerve axon density. **a** An exemplary profile of 12 iOHT eyes conducted at the ninth day of the experiment shows the effective mean IOP (ordinate) during simulation of a 1-h wavelike IOP profile (abscissa). IOP reduces to 10 mmHg after anesthesia, and IOP is raised to 35 mmHg for 20 min (interval I), followed by 45 mmHg for another 20 min (interval II). The loop was released to simulate a pressure drop. IOP was reset to 35 mmHg for 20 min (interval III), and IOP decreased to 12.5 mmHg during recovery phase. *Arrows* indicate important pressure changes before and after iOHT. **b** The survival of optic nerve axons was evaluated as the number of axons in an area of 0.05 mm^2^. Injured eyes of iOHT group showed a significant loss of optic nerve axons compared with fellow eyes in the same group (*gray bar*, *p* < 0.01) and control eyes (*black bar*, *p* < 0.01). Additionally, axon densities of injured eyes of iOHT group were significantly reduced compared with fellow eyes of iOHT + Belimumab group (*grey bar*, *p* < 0.01) but not to injured eyes of the iOHT + Belimumab group (*horizontal lines*, *p* = 0.05). Note that no significant loss of axonal density was observed for injured eyes of the Belimumab-treated group compared with fellow eyes (*p* = 0.5) and with control eyes (*p* = 0.07). Significances are indicated as follows: **p < 0.01. **c** A representative p-phenylenediamine-stained ultrathin transverse cross-sections (×10 magnification) shows the area where 15 high-resolution pictures were taken for the optic nerve axon density analysis. In total, 32 % of the optic nerve was analyzed for axon density survival. *Scale bar* represents 200 μm. **d**–**h** Representative high-resolution images (×100 magnification) show different stages of degeneration in optic nerve cross-sections. Healthy axons were present in control optic nerves (ON, **d**), while fellow eyes of iOHT ON (**e**) and iOHT + Belimumab (**g**) showed a slight damage, including swollen and collapsing axons indicated by *arrows*. Major axon degeneration with bulged myelin sheaths around axons (*arrows*) and gliotic areas (*triangle*) were observed in injured ONs of iOHT group (**f**) and iOHT + Belimumab group (**h**). *Scale bar* represents 20 μm

In accordance with the previously described technique for minimally invasive short-term IOP elevations [19, 20], iOHT was conducted in a wavelike profile. Adjusting a silicone loop around the limbus of the right eye (Fig. 1a), it is possible to narrow the anterior iridocorneal angle without modifying the shape of the eye or inducing subsequent structural injuries to the eye, which was monitored by regular funduscopy. In contrast to the first description of this method by Joos [19], the loop was not placed posterior to the limbus, but around the limbus. The loop slightly compresses Schlemm’s canal and the episcleral veins (Fig. 1b’) and provokes a marginal relocation of the iridocorneal angle, resulting in an increase of aqueous humor outflow resistance. Rats were anesthetized using 0.185 ml/kg body weight medetomidine (Dorbene vet., Pfizer, New York, NY, intramuscular administration), and eyes received oxybuprocain (Novesine 0.4 %, OmniVision, Puchheim, Germany) prior to, and regularly during, the procedure. Fellow eyes were covered with dexpanthenol to avoid corneal dehydration and prevent superficial irritations (Bepanthen, Bayer, Leverkusen, Germany). Once the loop is attached around the eye, the IOP rises immediately but returns to baseline levels within minutes when no adjustment of the loop occurs [20]. If the mean IOP was lower than the desired level, the diameter of the loop was slightly decreased. Conversely, the diameter was increased by gentle release of the loop when the mean IOP was too high. To mimic IOP fluctuations, which is a common observation in glaucoma patients, a wavelike IOP profile was applied to the animal’s eyes. This profile consists of three intervals of 20 min to 35 mmHg (interval I), 45 mmHg (interval II), and again to 35 mmHg (interval III). The loop was removed for 10 min between intervals II and III to simulate a pressure drop to a physiologic IOP level (Fig. 2a). The IOP elevation was performed on five consecutive days followed by 2 days of rest for 5 weeks. In week 6, IOP was elevated on two consecutive days and animals were sacrificed afterwards. Thus, each animal received a total of 27 unilateral IOP elevations. One group received IOP elevation (iOHT), while another group received 10 mg/kg of the B lymphocyte inhibitor Belimumab (Benlysta, GlaxoSmithKline, Parma, Italy, intravenous administration), initially 3 days before the first iOHT and weekly throughout the study in addition to IOP elevations as described above (iOHT + Belimumab). Control animals underwent the same examinations in a corresponding time frame but were not subjected to iOHT injury (CTRL).

After the final in vivo experiments, all animals were euthanized by CO_2_ inhalation followed by cardiac blood withdrawal and cardiac perfusion with 4 % paraformaldehyde/heparin (PFA, 2000 U/l) solution (Histofix, Roth, Karlsruhe, Germany). Eyes were marked in the nasotemporal orientation, dissected, immersion-fixed in 4 % PFA, and embedded in paraffin. Optic nerves with the optic chiasm attached were harvested, preserved in 3 % glutaraldehyde/0.1 mol/l sodium cacodylate buffer (Merck, Darmstadt, Germany) and processed for epoxy resin embedding.

### Quantification of axonal loss in optic nerves

0.7 μm semithin transverse optic nerve sections, taken 2 mm distal to the optic chiasm, were stained with p-phenylenediamine (PPD), and quantification of the axon density was performed as described previously [20, 23]. Briefly, 15 images from one representative section per optic nerve were captured in a predetermined crosswise pattern at ×10 magnification (2 C). This sampling method covers 32 % of the total optic nerve section. The semi-automated algorithm for quantification of the axon density using ImageJ software (NIH, Bethesda, MD, USA) contained the following steps: (1) images were converted into 8-bit format; (2) the automated threshold setting was verified and corrected manually by an independent observer masked to the animals’ status; and (3) numbers of axons were finally determined using a macro for the particle analyzer. Axon density of optic nerve cross-sections is shown as number of axons/0.05 mm^2^.

### RGC analysis and immunedetection of IgG autoantibodies and microglia

For each staining procedure, five 10 μm sagittal sections per eye were dewaxed, rehydrated, and blocked for endogenous peroxidase in 0.5 % H_2_O_2_ solution for 30 min. RGC labeling, microglia, and immunoglobulin G (IgG) autoantibody detection were conducted in accordance with routine procedures [24, 25]. Brain-specific homeobox/POU domain protein 3A (Brn3a) immunostaining was used as a RGC marker [26, 27]. Slides underwent antigen retrieval in target retrieval solution (TRS, DAKO, Hamburg, Germany) for 50 min at 70 °C and blocked in 2 % BSA/ 0.1 % Triton X-100/PBS solution (all Sigma) for 60 min. A goat anti-Brn3a antibody (C20, St. Cruz Biotechnology, Santa Cruz, CA, USA) was diluted 1:400 in blocking buffer, and specimens were incubated overnight at 4 °C. Sections were washed in PBS, and a horseradish peroxidase (HRP) conjugated rabbit anti-goat IgG (DAKO) diluted 1:400 in PBS was applied for 3 h at room temperature.

For immunolabeling of ionized calcium binding adaptor molecule 1 (Iba1), slides were blocked with 0.25 % NGS/0.5 % BSA/0.1 % Triton X-100/PBS after TRS antigen retrieval for 30 min at 90 °C. Rabbit anti-Iba1 (WAKO chemicals GmbH, Neuss, Germany, 1:5000) primary antibody was diluted in 0.1 % Triton X-100/PBS. After overnight incubation at 4 °C, sections were washed in PBS followed by goat anti-rabbit IgG (H + L) chain specific HRP-conjugated (Calbiochem, Merck KGaA, 1:1000, 60 min) secondary antibody incubation.

For IgG autoantibody staining, slides were boiled in TRS solution for 45 min at 90 °C and blocked in 1 % BSA/0.1 % Triton X-100/PBS solution for 10 min. A goat anti-rat IgG HRP-conjugated antibody (Thermo Scientific, Waltham, MA, USA) was diluted 1:500 in blocking buffer, and sections were incubated overnight.

After several wash steps in PBS, 3,3′-diamicobenzidin (DAB, DCS diagnostics, Hamburg, Germany) incubation for 10 min followed for color development. All sections were counterstained with hematoxylin, dehydrated, and coverslipped. For image acquisition, an Eclipse TS 100 microscope (Nikon, Yurakucho, Tokyo, Japan) with a DS-Fi1-U2 digital microscope camera (Nikon) and an ELWD 20x/0.45 S Plan Flour Ph1 ADM objective (Nikon) was used and recorded by the imaging software NIS Elements (Nikon, Version 4.10 64 bit). Images were transferred to CorelDRAW X4 Graphic Suites (Corel Corporation, Ottawa, Canada).

Data for each parameter was obtained from four representative cross-sections for each eye, containing the optic nerve head to ensure corresponding retinal position. The number of cells is calculated per 1-mm retinal length.

### Determination of serum IgG concentration

Blood samples from the cardiac blood withdrawal were allowed to clot at room temperature and were centrifuged at 10 °C for 10 min and 4000 rpm. Individual serum IgG concentrations of all animals were determined using a rat IgG ELISA kit (Genway, San Diego, CA, USA) according to the manufacturer’s protocol. Remaining serum samples were stored at −20 °C for further analysis of the antibody pattern.

### Antigen microarray assay

Using a non-contact array spotter (sciFLEXARRAYER 3, Scienion, Berlin, Germany), ten antigens (Additional file 1: Table S1) of interest were spotted in triplicates on nitrocellulose covered glass slides (Oncyte, nitrocellulose 16 multi-pad slides, Grace Bio-Labs, Bend, OR, USA). Blocking (0.5 % bovine serum albumin (BSA, Sigma, Steinheim, Germany) of unspecific binding sites of the nitrocellulose membrane in 0.5 % Tween 20 in phosphate buffered saline, (PBS-T, Gibco Life Technologies, Carlsbad, CA, USA) was performed for 1 h. Slides were incubated with animal’s serum taken at the end of the study (1:100 in PBS), and the assay was run as described previously [28]. Resulting antigen-antibody complexes were visualized by the use of the secondary antibody goat anti-rat IgG (H + L) Cy5 (Invitrogen, Carlsbad, CA, USA; 1:250 in PBS) Finally, slides were spin-dried (SpeedVac Concentrator 5301; Eppendorf, Hamburg, Germany), and emitted fluorescence signals were scanned using a high-resolution confocal array scanner (Affymetrix 428, Santa Clara, CA, USA).

For image and data processing, digitized signals (TIFF files) were analyzed with Imagene 5.5 (BioDiscovery Inc., Los Angeles, CA, USA). Technically faulty spots were identified by visual inspection and removed from the data set. For each spot, the local background intensity was subtracted from the median spot intensity. Intensity of the three technical replicates for each antibody was averaged and used for statistical analysis.

### Statistical analysis

For multiple test schemes, Tukey’s HSD post hoc (unequal N) tests were used. Comparison between two groups was performed using student’s independent *t* test. Data mining of microarray experiments was performed using one-way ANOVA and post hoc testing [28]. The correlation coefficient r was determined correlating the number of axons and the individual relative intensities per antigen. *P* values <0.05 were considered significant. Data are given as mean ± standard deviation (SD) or as group median. All statistical analyses were performed by Statistica Version 12 (Dell Inc. Round Rock, TX, USA).

## Results

### IOP in awake rats and exposure to elevated IOP

The IOP values of awake animals stayed within the normal physiological range of 10 and 13.3 mmHg and displayed no significant changes throughout the study (Additional file 2: Table S2). Additionally, IOP was recorded during procedures of iOHT in anesthetized animals. These IOP data were cumulated to display the total ΔIOP exposure in injured eyes over the fellow eyes [20, 29]. In total, 27 wavelike, 60 min-long IOP elevations led to a ΔIOP exposure of 748 ± 12 mmHg. The treatment with Belimumab did not interfere with the induction of iOHT and revealed a similar ΔIOP exposure of 756 ± 20 mmHg (*p* = 0.26).

### Intermittent IOP elevation alters optic nerve axon numbers and RGC density

Analysis of the axon density in the optic nerves revealed a loss of −11.9 % (*p* < 0.001) in iOHT injured eyes and −1.5 % in iOHT fellow eyes compared with control eyes. Belimumab-treated animals showed axonal loss of −1.9 % in iOHT + Belimumab fellow eyes and −7.5 % in iOHT + Belimumab injured eyes compared with control eyes. Injured eyes of the iOHT + Belimumab group revealed a slightly higher axonal density (+4.9 %) compared with iOHT group, even though this was not statistically significant (independent *t* test: *p* = 0.12, Fig. 2b, Table 1).

**Table 1:** Summary of the densities of the axons, retinal ganglion cells, microglia, and IgG depositions

Group	Number of axons per 0.05 mm^2^ ± SD (*n* of optic nerves)	*p* vs control	*p* vs iOHT injured eye	Brn3a-positive cells per mm retina ± SD (*n* of eyes)	*p* vs control	*p* vs iOHT injured eye	Iba1-positive cells per mm retina ± SD (*n* of eyes)	*p* vs control	*p* vs iOHT injured eye	IgG depositions per mm retina ± SD (*n* of eyes)	*p* vs control	*p* vs iOHT injured eye
Control eyes	22,267 ± 1408 (12)		*0.0003*	95.2 ± 12.6 (13)		*0.0001*	5.2 ± 1.1 (14)		*0.01*	0.5 ± 0.4 (14)		*0.002*
iOHT fellow eye	21,943 ± 1510 (12)	0.98	*0.001*	82.0 ± 9.9 (11)	0.07	*0.02*	6.7 ± 1.3 (10)	*0.03*	1	1.1 ± 0.4 (10)	*0.045*	0.75
iOHT injured eye	19,624 ± 1709 (12)	*0.0003*		66.0 ± 13.2 (11)	*0.0001*		6.7 ± 1.3 (12)	*0.01*		1.3 ± 0.6 (10)	*0.002*	
iOHT + Belimumab fellow eye	21,838 ± 802 (9)	0.96	*0.008*	80.9 ± 10.0 (9)	0.08	0.06	6.6 ± 1.0 (9)	0.08	0.99	0.9 ± 0.2 (9)	0.3	0.3
iOHT+ Belimumab injured eye	20,593 ± 609 (9)	0.07	0.5	74.2 ± 9.5 (8)	*0.005*	0.6	6.5 ± 1.0 (8)	0.1	0.99	0.9 ± 0.4 (8)	0.3	0.4

Analysis after 27 treatments using intermittent ocular hypertension shows the number of axons per 0.05 mm^2^ for all groups. These data illustrate the damage of optic nerves, and the number of Brn3a-positive cells indicate the overall density of retinal ganglion cells. Furthermore, the Iba1-positive cells imply the activation of microglia and immunoglobulin G (IgG) depositions, displaying the occurrence of autoantibody depositions. Brn3a, Iba1, and IgG data are given as total cell numbers per mm retina ± standard deviation (SD), and the number of animals in the analyses (*n*) are displayed. Statistically significant *p* values are highlighted in italics

Optic nerves obtained from untreated control animals and from uninjured fellow eyes showed minimal signs of neurodegeneration, such as swollen myelin sheaths, ruptured axons or gliosis (Fig. 2d, e, and g). Frequent axonal damage and some gliotic formations were only observed in optic nerve sections in eyes which underwent iOHT (Fig. 2f, h), independent of whether animals were without medication or received Belimumab.

**Fig. 3:**
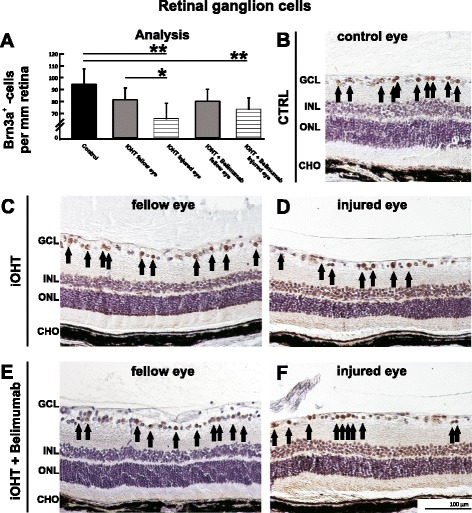
Retinal ganglion cell analysis. **a** The survival of retinal ganglion cells was analyzed as the number of Brn3a-positive cells per mm retina in naso-temporal cross-sections of the eye. A significant loss of retinal ganglion cells was observed in injured eyes of iOHT group and iOHT + Belimumab group (both *p* < 0.01, *horizontal lines*) compared with control eyes (*black*). Furthermore, a significant difference in damage was observed between fellow and injured eye of iOHT group (*p* < 0.05), but not between both eyes of iOHT + Belimumab group (*p* = 0.8). Significant values are indicated as follows: *p < 0.05, **p < 0.01. **b**–**f** Representative images of retinal cross-sections (×20 magnification) show different densities of Brn3a-positive cells (*arrows*) in controls (CTRL, **b**), fellow and injured eyes of iOHT group (**c** and **d**, respectively), and fellow and injured eyes of iOHT + Belimumab group (**e** and **f**, respectively). Images were taken at the same eccentricity to the optic nerve head to ensure comparability. Note the low number of retinal ganglion cells in injured eyes. Abbreviations: *GCL* ganglion cell layer, *INL* inner nuclear layer, *ONL* outer nuclear layer, *CHO* choroid. *Scale bar* represents 100 μm. Significant values are indicated as follows: *p < 0.05, **p < 0.01

Examination of the RGC density recapitulated the results of the optic nerve axon analysis. Eyes of control animals (Fig. 3b) and those exposed to iOHT displayed normal retinal morphology (Fig. 3c–f), but a significant loss of −19 % of RGC compared with the RGC density of their fellow uninjured eyes (*p* = 0.02), or to RGC numbers of control animals (−30.5 %, *p* = 0.0001, Fig. 3a, Table 1). Additionally, contralateral eyes of the iOHT group showed a slight, but not significant, loss of RGC compared with controls. Belimumab-treated animals displayed decreased RGC density in eyes after iOHT. As was the case for axonal density, the RGC loss was not statistically significant in comparison to the fellow eyes (*p* = 0.76).

### Morphometry and distribution of microglia and IgG autoantibody depositions in the retina

**Fig. 4:**
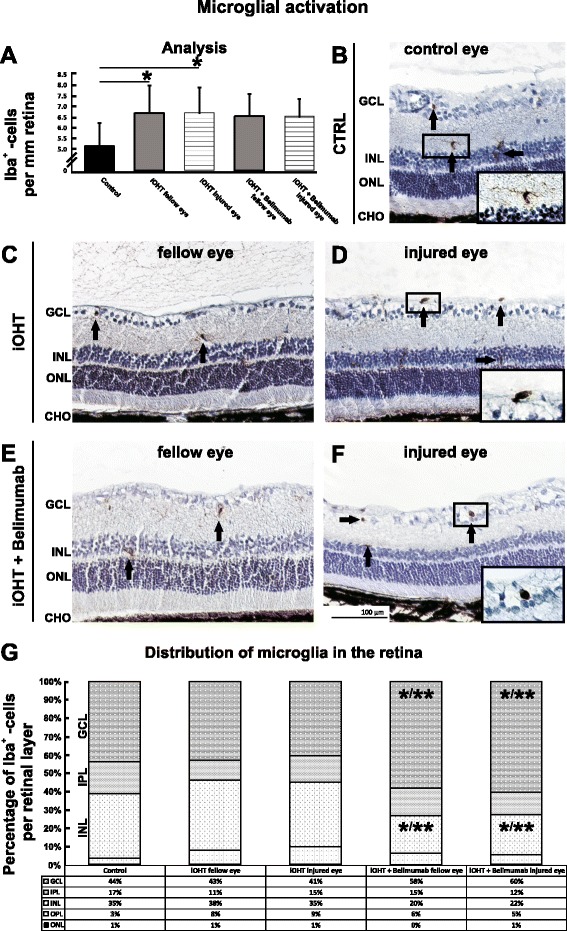
Activation of microglia and analysis of the distribution in the retina. **a** The activation of microglia was analyzed as Iba1-positive cells per mm retina in naso-temporal cross-sections of the eye. After iOHT, a significant increase of microglia occurred in fellow and injured eyes (both *p* < 0.05) compared with control eyes. No significant changes were observed for Belimumab-treated animals. Significant values are indicated as follows: *p < 0.05. **b**–**f** Representative images of retinal cross-sections (×20 magnification) show the numbers and distribution of Iba1-positive cells (*arrows*) in diverse retinal layers in control (CTRL, **b**), fellow and injured eyes of iOHT group (**c** and **d**, respectively), and fellow and injured eyes of iOHT + Belimumab group (**e** and **f**, respectively). Magnifications of representative microglia show a transformation from non-activated ramified phenotype with long ramifications in controls (**b**) to an activated, amoeboid phenotype in injured eyes (**d**, **f**). **g** Percentile distribution of microglia cells in retinal layers are given for all groups. Data were normalized in relation to the difference of the total number of microglia between the groups. Significant values are indicated as follows: *p < 0.05, **p < 0.01. *Abbreviations*: *GCL* ganglion cell layer, *IPL* inner plexiform layer, *INL* inner nuclear layer, *OPL* outer plexiform layer, *ONL* outer nuclear layer, *CHO* choroid. *Scale bar* represents 100 μm

To detect activation of retinal microglia, immunohistological staining of retinal cross-sections was performed and showed the occurrence of Iba1-positive cells in almost all retinal layers. In comparison to the control group, the amount of Iba1-positive cells was significantly higher in the eyes of the iOHT group (*p* < 0.03) and virtually identical to numbers found in animals which received iOHT + Belimumab treatment (*p* = 0.99, Fig. 4a, Table 1). The majority of microglia cells in the eyes of the iOHT groups that were located in the ganglion cell layer displayed an amoeboid phenotype (Fig. 4c–f, inset), while microglia in controls showed a ramified morphology with high numbers of long and thin processes (Fig. 4b, inset). Likewise, microglia cells of the inner plexiform layers and outer retinal layers exhibited a uniform, ramified morphology in all groups.

When determining the percentile distribution of microglia in retinal layers, the main appearance of microglia in control eyes was in the ganglion cell layer (44 %) as well as in the inner nuclear layer (35 %). Despite an increase of the total number of microglia in the iOHT group, their distribution was as observed in control eyes. In the iOHT + Belimumab group, a larger fraction of microglia was found in the ganglion cell layer of the injured eye (60 %), as well as the uninjured control eyes (58 %, *p* < 0.01 compared with control eyes and iOHT injured eyes and *p* < 0.05 compared with iOHT fellow eyes). Conversely, a significant reduction of microglia in the inner nuclear layer to 20 and 22 % in Belimumab-treated eyes occurred compared with iOHT fellow eyes (*p* < 0.01) or to iOHT injured eyes (*p* < 0.05). Apparently, a redistribution of microglia from the inner nuclear layer to the ganglion cell layer occurred in the eyes of Belimumab-treated animals (Fig. 4g, Additional file 3: Table S3).

**Fig. 5:**
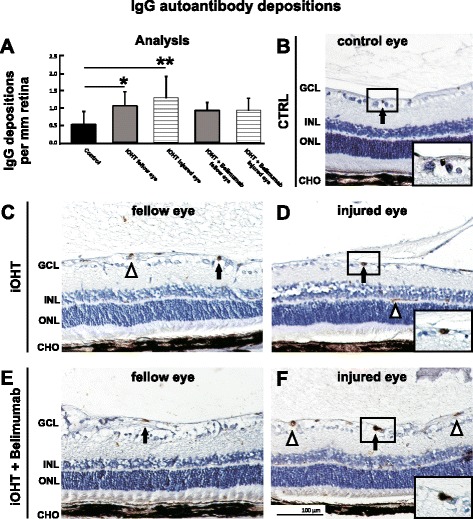
Analysis of IgG autoantibody depositions. **a** Autoantibody depositions were analyzed as immunoglobulin G (IgG)-positive depositions per mm retina in naso-temporal cross-sections of the eye. A significant increase of IgG autoantibody depositions could be observed in fellow and injured eyes of the iOHT group (*p* < 0.05 and *p* < 0.01, respectively) compared with control eyes, but not in iOHT + Belimumab group. Significant values are indicated as follows: *p < 0.05, **p < 0.01. **b**–**f** Representative images of retinal cross-sections (×20 magnification) show distinct IgG-positive depositions (*arrows*) in the ganglion cell layer of the retina. Magnifications highlight the dense morphology of these depositions (**b**, **d**, and **f**). *Triangles* indicate IgG-positive blood vessels containing erythrocytes, which must not be confused with IgG depositions. *Abbreviations*: *GCL* ganglion cell layer, *INL* inner nuclear layer, *ONL* outer nuclear layer, *CHO* choroid. *Scale bar* represents 100 μm. Significant values are indicated as follows: *p < 0.05, **p < 0.01

The analysis of retinal IgG autoantibody depositions revealed twice the amount of autoantibodies in the eyes of iOHT animals compared with controls and a more modest increase of autoantibody deposits in eyes of animals that received Belimumab (Fig. 5a, Table 1). The majority of these were located in the ganglion cell layer, and extravasal IgG autoantibody depositions were easily identified based upon their dense formations in the absence of blood vessel. No IgG autoantibody depositions were observed in the outer nuclear layer. In comparison to control eyes with 0.5 ± 0.4 IgG autoantibody depositions per mm retina, the number of IgG autoantibody depositions was significantly higher in eyes that underwent iOHT (1.3 ± 0.6, *p* = 0.002) as well as in fellow eyes (1.1 ± 0.4, *p* = 0.045). Animals that received Belimumab showed higher, though not statistically significant, levels of IgG autoantibody depositions in iOHT eyes (0.9 ± 0.4, *p* = 0.3) as well as in their fellow eyes (0.9 ± 0.2, *p* = 0.3, Fig. 5b–f) compared with control eyes.

### Influence of IOP elevations on the humoral immune response

The serum IgG titer of each group was quantified by ELISA. Control rats at the age of 14 weeks had a mean IgG titer of 2.55 ± 0.66 ng/ml. iOHT animals showed a similar level of IgG at 2.74 ± 0.38 ng/ml (*p* = 0.72). The administration of Belimumab significantly reduced the IgG titer to 1.80 ± 0.24 ng/ml in comparison to control animals (*p* = 0.02) and to iOHT-treated animals (*p* = 0.003).

**Fig. 6:**
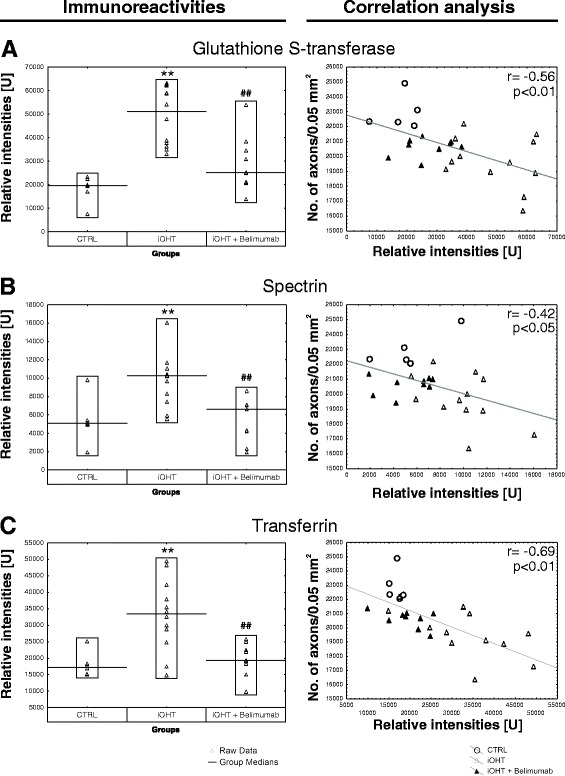
Systemic immune response. Quantification of different antigen reactivities of control animals (CTRL), animals that received unilateral intermittent ocular hypertension (iOHT), and animals with unilateral intermittent ocular hypertension which received Belimumab treatment (iOHT + Belimumab). The left side (**a**–**c** )shows the immunoreactivities in relative intensities [U] per group against glutathione S-transferase (**a**), spectrin (**b**), and transferrin (**c**). *Each triangle* represents one animal, and the *black line* indicates the group median. Compared with the relative intensity of CTRL, iOHT was significantly upregulated (**, *p* < 0.01) for all investigated antigens. Compared with iOHT, all immunoreactivities of iOHT + Belimumab were significantly downregulated ^(##^ *p* < 0.01). All *p* values were calculated by Tukey’s HSD post hoc test (unequal N). Scatterplots of the number of optic nerve axons in a distinct area (no. of axons/0.05 mm^2^) against relative intensities of glutathione S-transferase (**a**), spectrin (**b**), and transferrin (**c**) are shown on the right side. For correlation analysis, *p* values lower than 0.05 were considered as significant. The correlation coefficient *r* ranged between −0.42 and −0.69 for the investigated antibody reactivities (shown in upper right corner of the diagrams). The *gray fitting line* shows the negative linear dependence between the number of axons per 0.05 mm^2^ and the relative intensities. CTRLs are represented as *empty circles*, iOHT as *empty triangle*, and iOHT + Belimumab as *filled triangle*. Significant values are indicated as follows: **p < 0.01 compared with control group, ^##^ *p* < 0.01 compared with iOHT group

Antigen microarray analysis revealed significantly altered IgG autoantibody reactivities against the antigens glutathione S-transferase (CTRL: 18,280 ± 5752 U, iOHT: 49,091 ± 12,184 U, iOHT + Belimumab: 29,301 ± 11,925 U), spectrin (CTRL: 5391 ± 2519 U, iOHT: 9876 ± 2850 U, iOHT + Belimumab: 5429 ± 2323 U), and transferrin (CTRL: 18,055 ± 3682 U, iOHT: 33,020 ± 10,789 U, iOHT + Belimumab: 19,680 ± 4974 U). All of these changes were statistically significant when compared between iOHT and controls (glutathione S-transferase: *p* = 0.0002, spectrin: *p* = 0.006, transferrin: *p* = 0.003). Treatment of animals with the B lymphocyte inhibitor Belimumab led to significantly decreased immunoreactivity compared with iOHT animals (glutathione S-transferase: *p* = 0.001, spectrin: *p* = 0.002, transferrin: *p* = 0.003, Fig. 6a–c, left row). Importantly, statistically significant changes of the autoantibody reactivity between animals that received Belimumab and controls were not observed.

A correlation analysis between the number of optic nerve axons and the relative intensities for specific antibody-antigen reactions was performed. The results showed that the immunoreactivity against glutathione S-transferase (*r* = −0.56, *p* = 0.003), spectrin (*r* = −0.42, *p* = 0.03), and transferrin (*r* = −0.69, *p* = 0.0001, Fig. 6a–c, right row) in iOHT animals were negatively correlated to their respective axon densities. Accordingly, lower immunoreactivities of antibodies against glutathione S-transferase, spectrin, and transferrin corresponded to higher axonal survival after iOHT.

## Discussion

This study allows the following six conclusions regarding retinal injury and the involvement of immune responses in this rat model of intermittent ocular hypertension:

(i) iOHT elicits neurodegeneration in the optic nerve and the retina, although the fluctuating IOP pattern does not have more destructive potential than constantly elevated IOP.
(ii) Retinas of contralateral, uninjured eyes display pathological changes in comparison to untreated control eyes.
(iii) Retinal microglia in iOHT eyes are enriched in the retinal ganglion cell layer and activated.
(iv) iOHT eyes accumulate IgG autoantibody depositions in the retina.
(v) Serum autoantibody reactivities against glutathione-S-transferase, spectrin, and transferrin significantly increased and might serve as biomarkers.
(vi) Repeated injections of the B lymphocyte inhibitor Belimumab have very little effect on neurodegeneration, deactivation of microglia, and reduction of IgG autoantibody depositions despite lowering the total IgG serum concentration.

The main risk factor for glaucoma is elevated IOP [1–3]. In this study, a silicone loop was attached around the limbus of the eye to temporarily increase the IOP. After the manipulation, the IOP values returned to baseline levels, which demonstrates that this model does not cause angle-closure glaucoma. In this glaucoma animal model, a total ΔIOP exposure of 748 to 756 mmHg was achieved during 27 IOP elevations without affecting the IOP of the contralateral fellow eye. Intermittent ocular hypertension in a wavelike profile causes neuroretinal degeneration, which was indicated by the loss of axonal density by −10.6 % in injured optic nerves and decline of Brn3a^+^ cells by −19.5 %. Interestingly, these findings are comparable to those from a previous study with similar parameters (duration, animals, experimental setup), but a static IOP elevation [20]. Even though the mean ΔIOP exposure (752 ± 16 mmHg; iOHT and iOHT + Belimumab) in this study is slightly higher than the ΔIOP exposure during the previous study (737 ± 10 mmHg), the rate of axonal loss in the optic nerves is similar in both studies (−17 %). Thus, a relationship between the fluctuating IOP patterns and the progression of RGC loss could not be proven. Degeneration of optic nerve axons, and RGC, seems to be primarily driven by IOP elevations themselves rather than by fluctuations of the IOP. In this model, neurodegeneration accounts for −10.6 % optic nerve axonal loss and −19.5 % RGC loss, which is comparable to another rat glaucoma model by Semdowski et al. [30]. In their model, polystyrene microbeads are injected intravitreally and lead to IOP levels between 20 to 40 mmHg over 6 weeks. Due to the higher cumulative IOP exposure, neurodegeneration accounts for −25 % optic nerve axonal loss and −34 % RGC loss.

### Immune response in the neural retina

Activation of retinal microglia is a characteristic finding in IOP-associated animal models of glaucoma [31–34]. Interestingly, a bilateral activation of microglia in the retina and optic tract after unilateral IOP elevation has been reported in several studies [32–35]. Likewise, a significantly increased amount of microglia not only in eyes after elevation of the IOP but also in contralateral, uninjured eyes was also observed in this study. The majority of microglia in retinas displayed a transformed amoeboid phenotype with a dense cell body and sparse but thick ramifications (Fig. 4d), indicating activation of these immunocompetent cells. Microglia function during retinal degeneration is controversial, and activation of microglia cells should not be equated to being detrimental for neuronal survival per se. Moreover, microglia activation includes self-regulatory mechanisms to counterbalance over-activation such as enhanced secretion of neurotrophic factors to lessen neuronal death and restore tissue homeostasis. In this scenario, late stage microglia activation in the retina, and especially in the contralateral eye, might potentially be neuroprotective [32]. On the other hand, early microglia response clearly seems to be associated with the initiation of neurodegenerative processes (reviewed in [36–38]). However, a question that remains unsolved is how the stimulus of microglial activation is transferred from the injured to the unaffected eye. One hypothesis suggests that degeneration of optic nerve axons in the lateral geniculate nucleus might affect the synaptic terminals of RGCs from the contralateral eye, possibly resulting in a retrograde transmission of the neurodegenerative stimulus [39]. Another possibility is that increased MHC-II expression and antigen presentation in the optic nerve head after iOHT leads to priming of T lymphocytes, which in turn are able to activate microglia cells in the contralateral eye [32]. In another study, the same authors demonstrate increased numbers of Iba1^+^/MHC-II^+^ cells and rare Iba1^+^/CD68^+^ cells in the contralateral eye, suggesting an activation of the “M1” microglial phenotype. However, a decline in RGC density in the contralateral eye could not be determined in this study [33]. The theory of an involvement of T lymphocytes to induce a proinflammatory stimulus to the retina has received additional support by a recent study in which T lymphocytes from glaucomatous animals were transferred into healthy recipients. The recipients demonstrate a mild activation of microglia cells and a significant loss of RGC [40]. All these studies indicate the involvement of the immune system in the pathology of RGC loss, but additional experiments are needed to investigate the mechanism of contralateral microglia activation after unilateral injury in more detail.

IgG autoantibodies occur naturally in the healthy retina, but an increased number of retinal IgG autoantibody depositions have been observed in human glaucomatous retina [18], animal models of experimental autoimmune encephalomyelitis [24], and autoimmune glaucoma [25, 41]. Analogously, significantly increased amounts of IgG autoantibody accumulations were found bilaterally in retinas after unilateral iOHT (Fig. 5). Some autoimmune diseases affecting the central nervous system such as systemic lupus erythematosus or multiple sclerosis (MS) feature a direct involvement of specific immunoglobulins [13]. In the case of MS, autoantibodies against proteins of the myelin sheath directly cause degeneration of the axon [42, 43]. It has also been shown that autoantibodies act as part of the classical complement pathway and are involved in the pathobiology of AD (reviewed in [44]) and that specific autoantibodies against amyloid-ß deteriorate neuronal function [45]. These findings clearly demonstrate the harmful role of autoantibodies in neurodegenerative disorders. In glaucomatous subjects, as well as in several animal models of glaucoma, activation of the complement system has been shown [46, 47], but it has not been demonstrated that autoantibodies and C1q are co-located in the retina. Furthermore, a recent paper describes that the absence of immunoglobulins neither inhibits binding of C1q nor prevents RGC loss in an animal model of glaucoma [23]. These data do not support the argument that autoantibody accumulation in glaucoma is detrimental, and it might also be possible that some immunoglobulins found in glaucoma have beneficial functions (reviewed in [10, 11]). Unfortunately, the nexus on the function of immunoglobulins found in glaucomatous retina cannot yet be determined unequivocally.

### Humoral immune response

The overall aim of this study was the question whether or not a systemic immune response is initiated by RGC loss due to increased IOP and, more precisely, if the humoral immune response is correlated with optic nerve axon loss. Manipulation of the IOP elicits a significant increase of immunoreactivities against glutathione S-transferase, spectrin (alpha-fodrin, respectively), and transferrin compared with control animals. Likewise, an increased immunoreactivity of these autoantibodies, except transferrin, has been described for patients suffering from glaucoma [48, 49], indicating the close analogy of this experimental animal model to glaucoma. More interestingly, these three proteins are found to be involved in the pathobiology of other neurodegenerative diseases.

Glutathione S-transferase (GST) belongs to a family of multifunctional enzymes that act alone or along with enzymes within most mammalian organs and their cells to reduce superoxide radicals, hydroxyl radicals, and peroxynitrites [50]. The occurrence of GST protein in the human retina is thought to be neuroprotective [51, 52]. A study demonstrated that serum antibodies against the GST antigen are present in 52 % of patients with either primary open angle glaucoma or normal tension glaucoma. The occurrence of serum GST autoantibodies might be explained as an epiphenomenal event due to neurodegenerative processes in retinal ganglion cells undergoing apoptosis [48].

Spectrin is a member of the F-actin-crosslinking protein superfamily and is equivalent to alpha-fodrin [53]. This large, cytoskeletal and heterodimeric protein is involved in the cell cycle, cell adhesion, cell spreading, DNA repair, and intracellular traffic. Spectrin serves as a substrate of caspase-3 and calpain in neurons, leading to structural disorganization and membrane blebbing, an attribute of cells undergoing apoptosis [54]. Interestingly, anti-spectrin antibodies are suspected to be involved in the pathogenesis of a variety of neurodegenerative and autoimmune diseases such as AD [55], Sjögren syndrome [56], MS, and normal tension glaucoma [49], where elevated anti-spectrin immunoreactivities could be shown. During optic neuritis, the most common initial symptom of MS, higher levels of calpain led to elevated levels of spectrin cleavage [57]. It is therefore conceivable that the occurrence of serum antibodies against spectrin might be a result of major cleavage of spectrin in autoimmune diseases.

Transferrin is a serum iron-binding protein that is produced in the liver. It interacts with the transferrin receptor located on the cellular surface for delivery of redox active iron (Fe^3+^) molecules into the cell via receptor-mediated endocytosis in a pH-dependent manner [58]. Lower levels of transferrin after the initiation of inflammation due to infection, tissue injury, trauma, or immunological disorders lead to low levels of serum iron, which prevents microbial growth and is therefore beneficial for the infected organism [59]. In AD, the iron homeostasis dysregulation might lead to intracellular iron accumulation and to a rise of oxidative stress [60]. Very little data exists regarding the role of anti-transferrin antibodies in neurodegenerative diseases. However, in this study, elevated levels of anti-transferrin antibodies were identified, which might be an indicator for elevated serum levels of transferrin protein.

Moreover, the correlation analysis highlights that the loss of axons in the optic nerve in vivo is linked to the humoral immune response: The higher the immunoreactivity of autoantibodies against GST, spectrin, and transferrin, the lower axonal survival.

### Effects of B lymphocyte inhibition on RGC survival

Glaucoma is a complex disease with many contributing pathogenic factors apart from elevated IOP. To better understand the impact of the autoantibodies in glaucoma, Belimumab, an inhibitor of the B lymphocytes activation, was used in this study. Belimumab-treated animals did not affect IOP elevation, and treated rats were subjected to a similar ΔIOP than rats without medication. Although slightly higher numbers of axons and RGCs were detected in injured eyes of Belimumab-treated animals compared with the iOHT group, the differences are not statistically significant. It is of interest that the Belimumab-treated group displayed similar numbers of Iba1^+^ cells in the retina compared with the iOHT group (Table 1), but the distribution of microglia cells within the retinal layers was significantly different. The majority of microglia cells in this group was found in the ganglion cell layer, whereas a decreased number was observed in the inner nuclear layer of fellow and injured eye in iOHT + Belimumab-treated animals. It remains unclear if the increase of the retinal microglia population is related to retinal infiltration of immune cells. Iba1 immunostaining does exclusively label not only microglia but also monocytes [61], and it is not possible to distinguish between retinal microglia and infiltrating cells by Iba1 immunostaining. It is therefore also conceivable that microglia relocate to the ganglion cell layer in this group. Changes in the distribution of local microglia upon activation have been described for mouse models of age-related macular degeneration (AMD) [62] and glaucoma [63]. Based upon the observation that the shift in the microglial distribution occurred bilaterally leads to the assumption that this might be a result of the treatment with Belimumab. Nevertheless, Belimumab treatment is not sufficient to ameliorate optic nerve axon loss after iOHT, at least at a dosage of 10 mg/kg, but results in little improvement in the number of surviving optic nerve axons. It remains unclear if this marginal rescue effect is directly mediated through the drug itself or if it is a result of the increased microglial activity in the ganglion cell layer. Furthermore, injured eyes of Belimumab-treated animals showed slightly elevated numbers of IgG autoantibody depositions compared with control and slightly lower number compared with iOHT injured eyes. However, those differences were also not statistically significant (Table 1). As expected, Belimumab significantly decreased the IgG titer in iOHT + Belimumab animals demonstrating the drug’s bioavailability. Likewise, the immunoreactivity against GST, spectrin, and transferrin was also significantly decreased compared with iOHT group. Overall, the treatment with Belimumab in this study was not sufficient to significantly affect RGC and axonal loss, decrease microglial activation, and reduce IgG autoantibody depositions. Thus, the role of autoantibodies in the induction of glaucomatous damage in this experimental glaucoma model seems limited. On the other hand, changes in the reactivity of theses autoantibodies are clearly connected to RGC loss, and therefore, the autoantibody profiling may serve as an important biomarker for glaucoma [10, 11].

In summary, the question about the origin of the described antibody immunoreactivities in the iOHT group should be raised. To date, it remains elusive whether the elevated antibody reactivities in this animal model emerge due to recurring pressure fluctuations or if they are a consequence of the neuronal degeneration of RGCs and their axons. This intriguing question needs to be the subject of further studies to understand the role of autoantibodies in the pathogenesis not only of glaucoma but also of other neurodegenerative diseases such as AD, PD, and Amyotrophic Lateral Sclerosis.

## Conclusions

Unilateral intermittent IOP elevations led to a significant decrease of RGCs and their axons in the optic nerve, accompanied by bilateral microglia activation and IgG autoantibody depositions in the retina. Furthermore, a systemic immune response due to RGC loss in association with an increased IOP could be shown for the iOHT group with a significant increase of serum IgG reactivities as well as IgG immunoreactivities against glutathione S-transferase, spectrin (alpha-fodrin, respectively), and transferrin. Immunomodulation using the BAFF-blocker Belimumab to hinder B lymphocyte activation led to a significant decrease of serum IgG concentration, a lower glutathione S-transferase-, spectrin-, and transferrin immunoreactivities but shows only limited ability to ameliorate the progression of axonal and RGC loss. This result limits the detrimental role of autoantibodies in inducing glaucomatous RGC loss but underlines the importance of changes of autoantibody reactivities as a valuable biomarker. The present study gives insight into the close relationship between IOP, the immune response, and the pathology of RGC loss. Nevertheless, more research is needed in the future to understand the origin of an altered humoral immune response with focus on cellular interactions in glaucoma and in other neurodegenerative diseases.

## Ethics approval

Animals were treated according to the ARVO Statement for the Use of Animals in Ophthalmic and Vision Research, and all experiments have been approved by the national investigation office in Koblenz, Germany (23 177-07/G 11-1-029).

## Additional files

Additional file 1: Table S1. Summary of antigens used in the microarray experiments. Antigens of interest were spotted on microarray slides and incubated with serum to identify changes of immunoreactivities. Name, order number, and company are listed. (XLSX 10 kb) Additional file 2: Table S2. Course of IOP in awake Long Evans rats. The IOP of awake Long Evans rats stays in physiological ranges, and statistical analysis reveals consistent IOP in the control group as well as in fellow and injured eyes of the experimental groups at each time point (*p* < 0.02). The data shows no spatiotemporal increases of IOP as a considerable effect of multiple intermittent ocular hypertensions (iOHT). All IOP values are given as mean IOP in mmHg ± standard deviation (SD). (XLSX 10 kb) Additional file 3: Table S3. Summary of the distribution of microglia in the retinal layers. Microglia are distributed in diverse layers of the retina. In control eyes, most microglia occur in the ganglion cell layer (GCL) and the inner nuclear layer (INL). Eyes of iOHT group show no significant changes for microglia alterations between retinal layers. In contrast, a shifting of microglia populations can be observed from the inner nuclear layer to the ganglion cell layer in both eyes of Belimumab-treated animals. Data are given as mean number of Iba1-positive cells per mm retina ± standard deviation (SD). * indicates individual retinal layers without statistically significant changes. Statistically significant *p* values are highlighted in bold and red. (XLSX 11 kb)

## Abbreviations

AD: Alzheimer’s dementia
AMD: age-related macular degeneration
BAFF: B lymphocytes activating component of the tumor necrosis factor family
Brn3a: brain-specific homeobox/POU domain protein 3A
CHO: choroid
DAB: 3,3′-diamicobenzidin
GCL: ganglion cell layer
GST: glutathione S-transferase
HRP: horseradish peroxidase
Iba1: ionized calcium binding adaptor molecule 1
IgG: immunoglobulin G
INL: inner nuclear layer
iOHT: intermittent ocular hypertension
IOP: intraocular pressure
IPL: inner plexiform layer
MS: multiple sclerosis
ON: optic nerves
ONL: outer nuclear layer
OPL: outer plexiform layer
PBS: phosphate buffered saline
PD: Parkinson’s disease
PPD: p-phenylenediamine
RGC: retinal ganglion cell
SD: standard deviation
